# The role of dobutamine stress cardiovascular magnetic resonance in the clinical management of patients with suspected and known coronary artery disease

**DOI:** 10.1186/1532-429X-13-46

**Published:** 2011-09-12

**Authors:** Rolf Gebker, Cosima Jahnke, Robert Manka, Thomas Hucko, Bernhard Schnackenburg, Sebastian Kelle, Christoph Klein, Eckart Fleck, Ingo Paetsch

**Affiliations:** 1 German Heart Institute Berlin, Germany; 2 University Hospital RWTH Aachen, Germany; 3 Philips Health Care, Hamburg, Germany

## Abstract

**Background:**

Recent studies have demonstrated the consistently high diagnostic and prognostic value of dobutamine stress cardiovascular magnetic resonance (DCMR). The value of DCMR for clinical decision making still needs to be defined. Hence, the purpose of this study was to assess the utility of DCMR regarding clinical management of patients with suspected and known coronary artery disease (CAD) in a routine setting.

**Methods and Results:**

We prospectively performed a standard DCMR examination in 1532 consecutive patients with suspected and known CAD. Patients were stratified according to the results of DCMR: DCMR-positive patients were recommended to undergo invasive coronary angiography and DCMR-negative patients received optimal medical treatment. Of 609 (40%) DCMR-positive patients coronary angiography was performed in 478 (78%) within 90 days. In 409 of these patients significant coronary stenoses ≥50% were present (positive predictive value 86%). Of 923 (60%) DCMR-negative patients 833 (90%) received optimal medical therapy. During a mean follow-up period of 2.1 ± 0.8 years (median: 2.1 years, interquartile range 1.5 to 2.7 years) 8 DCMR-negative patients (0.96%) sustained a cardiac event.

In 131 DCMR-positive patients who did not undergo invasive angiography, 20 patients (15%) suffered cardiac events. In 90 DCMR-negative patients (10%) invasive angiography was performed within 2 years (range 0.01 to 2.0 years) with 56 patients having coronary stenoses ≥50%.

**Conclusion:**

In a routine setting DCMR proved a useful arbiter for clinical decision making and exhibited high utility for stratification and clinical management of patients with suspected and known CAD.

## Background

Physicians commonly have to determine the need for invasive angiography in patients with suspected or known coronary artery disease (CAD). Current guidelines and recent studies have emphasized the importance of non-invasive stress testing for the detection of myocardial ischemic reactions prior to invasive angiography [1-3]. Dobutamine stress cardiovascular magnetic resonance (DCMR) is well established for the evaluation of patients with suspected and known CAD [4-9]. Apart from merely detecting stress inducible myocardial ischemia, there is growing evidence supporting the value of DCMR to assess cardiac prognosis [10,11]. As for other imaging modalities, patients with an intermediate pretest probability for the presence of CAD benefit most from DCMR [12]. However, data on the usefulness of DCMR testing to direct patient treatment regarding medical versus invasive strategies has not been reported yet. Hence, we sought to evaluate the value of DCMR as the sole clinical decision maker in a large unselected patient population and determined its utility for clinical management of patients with suspected and known CAD.

## Methods

### Patient population

The study was conducted in accordance with the Institutional Review Board at the Charité University School of Medicine and written informed consent was given by all patients. Between November 2005 and July 2008, 1699 patients were examined prospectively for the evaluation of chest pain syndromes or dyspnea. Patients were eligible if they had suspected or known CAD including patients with prior interventional or surgical revascularization. Patients with contraindications to either CMR (non-compatible biometallic implants) or dobutamine (e.g. unstable angina, myocarditis, endocarditis) and patients with atrial fibrillation were not considered. All patients were instructed to refrain from beta-blockers 24 hours prior to the MR study. Medical history was obtained immediately before DCMR. Clinical variables were defined according to the Framingham Risk Score assessment [13].

### DCMR

As previously described DCMR was performed in the supine position on a 1.5 Tesla Intera CV system (Philips Medical Systems, Best, The Netherlands) [7]. Cardiac standard geometries (three short axis views and a four-, two- and three-chamber view) were acquired at rest and during dobutamine/atropine stress to achieve target heart rate, defined as 85% of the maximum predicted heart rate: (220-age) × 0.85. Termination criteria were as previously published [14]. Total examination time was ≈ 30 minutes.

### MR Sequences

For cine imaging, balanced steady-state free precession (bSSFP) sequences with retrospective gating (50 phases per cardiac cycle) were used during an end-expiratory breath hold [repetition time (TR), 3.4 ms; echo time (TE), 1.7 ms; flip angle, 60°]. In-plane spatial resolution was 1.8 × 1.8 mm with a slice thickness of 8 mm.

### Image analysis

The study sought to address the impact of DCMR on clinical management in routine practice, so the results of each study were interpreted at the time of the original examination by cardiologists trained in DCMR. All image analysis was performed on a commercially available View Forum station (Philips Medical Systems, Best, The Netherlands). Segmental analysis of wall motion was performed using a synchronized quad-screen image display and applying a standard 17-segment scoring system during each stage of the protocol (1 = normal, 2 = hypokinetic, 3 = akinetic, or 4 = dyskinetic) [15]. A positive DCMR (DCMR-pos) was defined as a stress inducible wall motion abnormality (IWMA) in ≥ 1 segment; a biphasic response was also considered to indicate a positive DCMR. A negative DCMR (DCMR-neg) was defined as the absence of a stress inducible wall motion abnormality. Left ventricular function was determined using a combined triplane model [16]. DCMR results were made available to the referring physician immediately after finishing the examination using a standardized reporting sheet.

### Follow-up

Outcome data were collected from a standardized mailed questionnaire, telephone interview with the patient or a close relative and hospital chart review; all reported clinical events were confirmed by contact with the patient's general practitioner or the treating hospital. Survival information was obtained from the Department of National Registration for patients lost on first contact. Cardiac death and nonfatal myocardial infarction were registered as cardiac events. Cardiac death was defined as death in the presence of acute coronary syndrome, fatal arrhythmia, refractory cardiac failure or sudden unexpected death; nonfatal myocardial infarction was defined by angina and an increase in cardiac-specific enzymes and/or development of new ECG changes (i.e., transient ST-segment elevation). In the case of 2 simultaneous events the worst event was chosen (cardiac death > myocardial infarction). We documented the reasons to perform invasive coronary angiography despite a negative DCMR during the 2 years following the examination with the period being chosen based on data from a previous prognosis study [11]. Conversely, the reasons not to perform invasive angiography within 90 days in patients with a positive DCMR were also noted. Patients who underwent coronary artery bypass grafting or percutaneous coronary intervention were censored at the time of revascularization.

### Coronary Angiography

Invasive coronary angiography was performed at the discretion of the referring physicians. The angiograms were evaluated visually for the presence of significant stenoses (i.e. ≥ 50% luminal diameter reduction) in the three large epicardial coronary arteries and their major branches (i.e. vessel diameter ≥ 2.0 mm) by highly experienced invasive cardiologists.

### Statistical analysis

Statistical analysis was performed using the SPSS software package release 15.0.1 (Chicago, USA). For all continuous parameters, data are expressed as mean ± standard deviation. Additionally, follow-up duration is presented as median with lower and upper quartiles. The normality of the distributions was tested with the Kolmogorov-Smirnov test. Comparisons between two groups of continuous data were made with unpaired Student t or Mann-Whitney U test where appropriate. Discrete data was analyzed with the chi-square and Fisher's exact test when appropriate. Sensitivity was calculated according to standard definitions. The Kaplan-Meier method was used to construct plots depicting clinical events as a function of follow-up duration, and curves were compared using the log-rank test. Statistical tests were two-tailed; significance was reached if p < 0.05.

## Results

### Patient Characteristics

Of the initial 1699 patients, 124 were excluded due to claustrophobia (n = 46, 2.7%), technical problems (e.g. ECG mistriggering and insufficient image quality) (n = 17, 1%) and limiting side effects during the administration of dobutamine/atropine with premature termination of the MR examination (n = 61, 3.6%), including 36 patients with severe chest pain and/or dyspnea, 20 with supraventricular tachycardia, 3 with severe increase in blood pressure > 240/120 mmHg and 2 with non-sustained ventricular tachycardia. None of the patients died or suffered from a myocardial infarction related to DCMR testing. Of the remaining 1575 patients, 43 were lost to follow-up (2.7%). Thus, final analysis was performed on 1532 patients. During DCMR target heart rate was achieved in 1442 patients (94%). Tables 1, 2, 3 and 4 provide the clinical baseline characteristics and hemodynamic data of the final patient population according to the result of DCMR, respectively.

**Table 1:** DCMR-positive vs.DCMR-negative patients

	DCMR-pos patients	DCMR-neg patients	
	
	N = 609	N = 923	p^†^
**Clinical characteristics**			
Age [years]	64 ± 9	62 ± 11	0.04
Male, %	74	63	<0.001
Body mass index [kg/m2]	28 ± 4	27 ± 4	<0.001
LVEF	56 ± 9	58 ± 6	<0.001
			
Hypertension, %	79	70	<0.001
Hyperlipidemia, %	75	59	<0.001
Diabetes mellitus, %	28	19	<0.001
Smoking, %	30	31	0.58
Family history, %	25	33	<0.001
Framingham risk score	16 ± 11	15 ± 11	0.04
			
Known CAD, %	68	35	<0.001
Prior myocardial infarction, %	35	29	0.03
Prior PCI, %	57	28	<0.001
Prior CABG, %	26	12	<0.001

Values are expressed as mean ± SD or %, ^†^DCMR-pos patients vs. DCMR-neg patients

**Table 2:** Clinical data in DCMR-pos patients

	**DCMR-pos patients**			**Patients with invasive angiography**		
		
	**invasive angio**	**no invasive angio**		stenosis*	no stenosis*	
	**n = 478**	**n = 131**	**p^†^**	**n = 409**	**n = 69**	**p^‡^**
	
**Clinical characteristics**						
Age [years]	64 ± 9	66 ± 9	0.03	64 ± 9	60 ± 10	0.001
Male, %	73	78	0.26	78	45	<0.001
Body mass index [kg/m2]	28 ± 4	27 ± 3	0.007	28 ± 4	28 ± 4	0.63
LVEF	56 ± 8	54 ± 11	0.61	55 ± 8	59 ± 6	<0.001
						
Hypertension, %	80	76	0.26	83	65	0.002
Hyperlipidemia, %	76	74	0.7	79	54	<0.001
Diabetes mellitus, %	29	24	0.31	30	20	0.11
Smoking, %	30	30	0.9	30	32	0.78
Family history, %	23	30	0.11	22	30	0.12
Framingham risk score	16 ± 11	18 ± 12	0.26	17 ± 11	12 ± 7	0.002
						
Known CAD, %	68	65	0.45	75	29	<0.001
Prior myocardial infarction, %	33	41	0.12	33	36	0.55
Prior PCI, %	58	53	0.34	64	26	<0.001
Prior CABG, %	26	27	0.9	30	6	<0.001
						
**Medication, %**						
Aspirin	93	95	0.51	98	56	<0.001
Betablocker	88	89	0.68	92	58	<0.001
ACE inhibitor	70	66	0.47	72	54	0.009
Angiotensin receptor blocker	26	30	0.36	25	29	0.61
Calcium channel blocker	32	30	0.74	31	39	0.34
Statin	92	90	0.64	96	58	<0.001
Diuretic	43	41	0.8	44	37	0.37

Values are expressed as mean ± SD or %, *luminal diameter reduction ≥50% on invasive coronary angiography, ^†^DCMR-pos patients with vs. without invasive coronary angiography,^‡^DCMR-pos patients with vs. without coronary stenosis

**Table 3:** Clinical data in DCMR-neg patients

	**DCMR-neg patients**			**Patients without invasive angiography**		
		
	**no invasive angio**	**invasive angio**		**no event**	**event**	
	**n = 833**	**n = 90**	**p^†^**	**n = 825**	**n = 8**	**p^‡^**
	
**Clinical characteristics**						
Age [years]	62 ± 11	64 ± 11	0.27	62 ± 11	59 ± 10	0.28
Male, %	62	72	0.06	62	71	0.61
Body mass index [kg/m2]	27 ± 4	27 ± 4	0.77	27 ± 4	28 ± 6	0.29
LVEF	58 ± 6	58 ± 8	0.31	58 ± 6	53 ± 14	0.76
						
Hypertension, %	70	76	0.26	70	86	0.36
Hyperlipidemia, %	57	71	0.01	57	86	0.13
Diabetes mellitus, %	19	22	0.39	18	29	0.49
Smoking, %	30	41	0.03	30	29	0.93
Family history, %	34	30	0.49	34	14	0.28
Framingham risk score	15 ± 11	16 ± 11	0.56	15 ± 11	18 ± 9	0.7
						
Known CAD, %	32	58	<0.001	32	57	0.15
Prior myocardial infarction, %	22	30	0.11	30	71	0.03
Prior PCI, %	25	52	<0.001	25	43	0.38
Prior CABG, %	10	22	0.001	10	29	0.16
						
**Medication, %**						
Aspirin	60	87	<0.001	60	86	0.25
Betablocker	62	76	0.01	61	100	0.04
ACE inhibitor	48	67	0.001	48	57	0.71
Angiotensin receptor blocker	25	20	0.4	25	14	0.53
Calcium channel blocker	21	27	0.28	21	14	0.65
Statin	57	87	<0.001	56	71	0.43
Diuretic	32	46	0.02	32	29	0.83

Values are expressed as mean ± SD or %, ^†^DCMR-pos patients with vs. without invasive coronary angiography, ^‡^DCMR-pos patients with vs. without coronary stenosis

**Table 4:** Hemodynamic data

	All patients	DCMR-pos	DCMR-neg	
	n = 1532	n = 609	n = 923	p^†^
**Hemodynamic data**				
Dobutamine dose (μg/kg/min)	35 ± 8	35 ± 8	35 ± 8	0.5
Atropine dose (mg)	0.25 ± 0.35	0.26 ± 0.36	0.24 ± 0.35	0.27
Resting HR, beats/minute	73 ± 13	72 ± 14	73 ± 13	0.008
Peak HR, beats/minute	139 ± 10	138 ± 11	140 ± 10	<0.001
Target HR achieved, %	94	91	97	<0.001
Resting SBP, mmHg	132 ± 22	133 ± 23	132 ± 22	0.68
Peak SBP, mmHg	141 ± 30	141 ± 32	141 ± 29	0.83
Resting Rate Pressure Product	9691 ± 2748	9607 ± 2829	9747 ± 2695	0.12
Peak Rate Pressure Product	18759 ± 4275	18893 ± 4351	18667 ± 4236	0.31

Values are expressed as mean ± SD or %, ^†^DCMR-pos vs. DCMR-neg patients

### Outcomes

**Figure 1:**
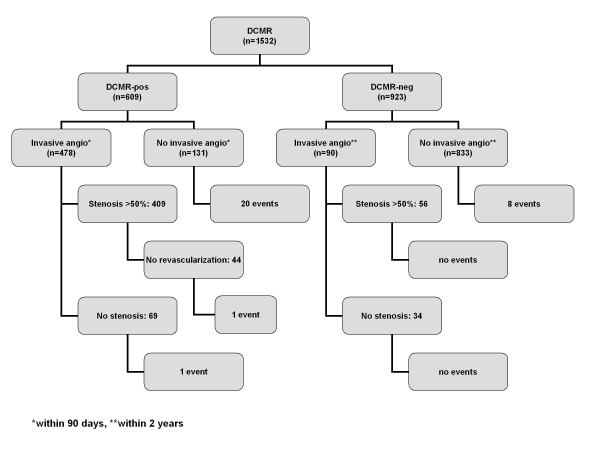
**Summarizes the outcome of our patient population according to the results of DCMR**.

**Figure 2:**
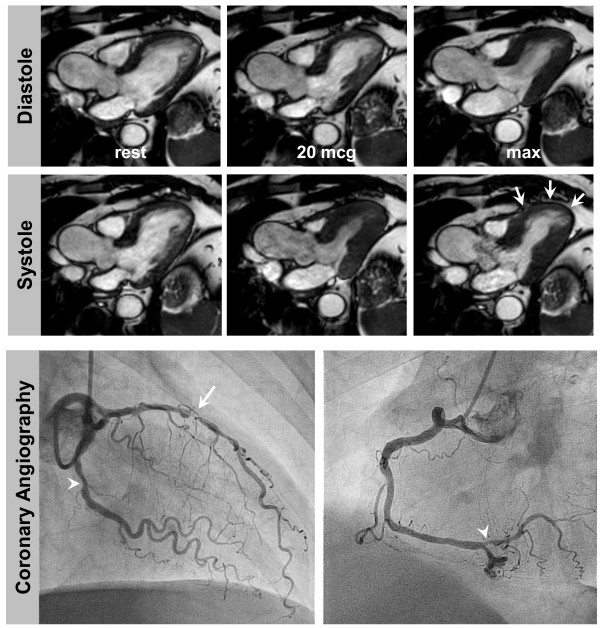
**DCMR in a 56 year old man with exertional dyspnoea and atypical chest pain**. He had arterial hypertension and was an active smoker without a prior history of CAD. He was referred for DCMR after a normal exercise ECG and insufficient image quality for a stress echocardiography. DCMR (top and middle) revealed a stress inducible wall motion abnormality of the apical and mid-ventricular anteroseptal segments (white arrows). Invasive angiography (bottom row) demonstrated high grade stenosis of the LAD (white arrow) and intermediate stenoses of the LCX and distal RCA (white arrowheads).

A summary of the outcome data is given in Figure 1. Table 5 provides a summary of events according to DCMR results. In 609 DCMR-pos patients elective invasive angiography was recommended. In 478 out of these 609 patients (78%) invasive angiography was performed within 90 days with 409 patients demonstrating hemodynamically relevant coronary stenoses ≥50% (positive predictive value 86%), see Figure 2 for an example of a DCMR-pos patient. Coronary revascularization was performed in 365 patients. In 69 DCMR-pos patients who underwent invasive angiography without demonstrating coronary stenoses 2 events occurred (event rate 2.9%); these patients had known CAD with moderately reduced LV-function. In 131 patients who did not undergo elective invasive angiography within 90 days 20 events (4 deaths and 16 myocardial infarctions) occurred (event rate 15.3%). The reasons not to perform elective invasive angiography despite a positive DCMR were: patient's refusal (n = 36, 27%; 5 events), few symptoms (n = 20, 15%; 1 event), limited amount of ischemia(n = 16, 12%; mean number of segments with IWMA 1.2 ± 0.4; 1 event), limited amenability to revascularization procedures (e.g. in known chronic coronary vessel occlusion (n = 17, 13%; 3 events)), early occurrence of an event within 90 days (n = 9, 7%) and remained unknown in 33 cases (25%; 1 event).

**Table 5:** Summary of events

	All	DCMR-pos	DCMR-neg	
	n = 1532	n = 609	n = 923	p^†^
**Events (%)**				
Cardiac death	8 (0.5)	5 (0.8)	3 (0.3)	0.28
Myocardial infarction	22 (1.5)	17 (3.0)	5 (0.5)	<0.001

Values are expressed as n (%), ^†^DCMR-pos vs. DCMR-neg patients

**Figure 3:**
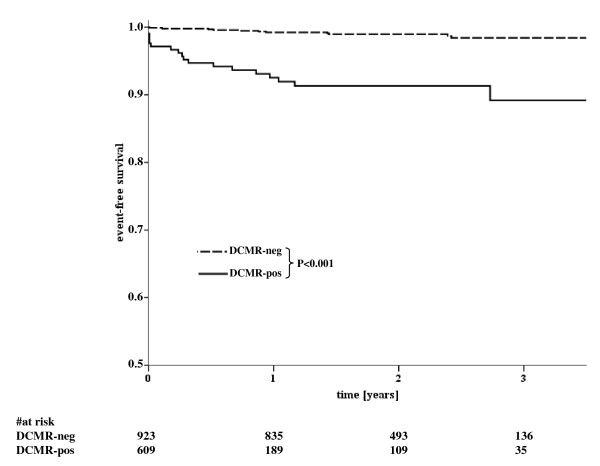
**Kaplan-Meier curves illustrating the time-to-event distributions of cardiac events stratified according to the results of DCMR testing**. Differences between the curves are statistically significant (P<0.001 by log-rank test).

In 923 DCMR-neg patients medical treatment was recommended. No elective invasive angiography was performed within 2 years in 833 out of these 923 patients (90%). Mean follow-up period was 2.1 ± 0.8 years (median: 2.1 years, interquartile range 1.5 to 2.7 years). In this patient subgroup 8 events (3 deaths, 5 myocardial infarctions) occurred (event rate 0.96%; Figure 3). Six out of these 8 patients (75%) had known CAD with prior sustained myocardial infarctions; the mean time to an event was 434 ± 219 days. In 90 DCMR-neg patients elective angiography was performed (10%) with a mean time to invasive angiography of 7 ± 7 months (median: 5 months, interquartile range 0.03 - 13 months), with 56 patients demonstrating coronary stenoses ≥50%. Out of these patients, 34 had single vessel CAD, 16 had double vessel CAD and 6 had triple vessel CAD, no patient had relevant left main disease. Coronary revascularization was performed in 46 patients. In the remaining 34 patients without coronary stenoses no events occurred during follow up. The reasons to perform elective invasive angiography within 2 years of a negative DCMR were: persisting complaints of angina/dyspnea (n = 46; 51%), pathologic findings on another stress test performed within 2 years (n = 21; 23%) or other clinical reasons (e.g. coronary angiography during an electrophysiologic study (n = 23; 26%)).

Table 6 demonstrates cumulative survival rates in patients stratified according to DCMR test results at 1-, 2- and 3-year follow-up intervals; the 3-year event-free survival was 98.5% for patients with negative DCMR and 94% for those with abnormal DCMR.

**Table 6:** Cumulative Survival Rates at Follow up Intervals

	Cumulative Survival Rates at Follow up Intervals, %		
Result of DCMR	1y	2y	3y
DCMR-negative	99,5	99,2	98,5
DCMR-positive	96,1	95,1	94,0

log Rank <0.001

### Segmental extent of ischemia vs. event rate

The mean number of segments with IWMA in the overall population was 1.18 ± 1.73. The number of segments with IWMA in patients with cardiac events was significantly higher compared to those without events (2.1 ± 1.8 vs. 1.2 ± 1.7, respectively; p = 0.003). In particular, patients with an early event occurring <90 days after DCMR showed a significantly higher number of segments with IWMA than those with a late event occurring >90 days after DCMR (3.42 ± 1.62 vs. 1.26 ± 1.41; p = 0.001).

## Discussion

The present study addressed the impact of DCMR on clinical management in a large unselected patient population with chest pain syndromes. The main findings are 1) DCMR is applicable in a clinical routine setting with a high success rate and few stressor related complications during a reasonably short examination time of less than 30 minutes, 2) DCMR proved useful as an arbiter for clinical decision making with regard to invasive versus medical treatment in patients with suspected and known CAD, 3) the positive predictive value of DCMR to detect coronary luminal narrowing >50% is high, 4) a positive DCMR is a powerful predictor of future cardiac events, and 5) a negative DCMR test result infers a low risk for subsequent cardiac events (about 1% in the two years after stress testing).

One of the most important clinical questions that non-invasive stress testing has to address is whether patients should be advised to undergo invasive angiography or to continue with medical treatment. As a result of major advances in imaging technology, several diagnostic strategies have become available over the past decades. Although exercise electrocardiography is advocated as a first-line procedure [1], sensitivity may be as low as 45 percent and many patients cannot exercise sufficiently due to poor functional status [17]. In order to determine a patient's most appropriate management multiple tests may be conducted and frequently yield conflicting results. Thus, in a large number of individuals a single imaging test in conjunction with pharmacological stress as the initial strategy may be the most effective approach in patient care. Whereas echocardiography and radionuclide imaging have been evaluated extensively, data on CMR based management strategies are scarce. DCMR has matured into a technically robust method with similarly high values for sensitivity and specificity of ≈ 85% for the detection of myocardial ischemic reactions in the presence of obstructive coronary lesions [12] and has been shown to provide relevant prognostic information [10,11,18]. Previous studies focused on the prognostic value of DCMR in low/intermediate versus high risk patient groups as defined by conventional cardiovascular risk factors and reported a relative merit of stress magnetic resonance testing [19]. The design of the present study, however, was unique in that it established the utility of DCMR testing as the sole clinical decision maker in a routine clinical setting: our study attributed DCMR testing an active role in clinical decision making with treatment directed either to a medical or invasive strategy. Consequently, while corroborating the usefulness of DCMR testing, our data closely reflects clinical reality in a tertiary care center setting and as such will be applicable to a similar clinical scenario.

The overall safety profile and frequency of adverse events of DCMR observed in our study are in agreement with previous reports using CMR and other well established methodologies using high dose dobutamine-atropine stress protocols [9,20]. Results of DCMR were communicated in a timely and definitive manner to the referring physician so that they formed the basis for subsequent clinical decision making.

In our study most patients with a positive DCMR underwent invasive angiography with the intention to perform revascularization. Compared to prior results regarding the diagnostic accuracy of DCMR, this study confirms the high predictive value for the detection of angiographically relevant obstructive coronary stenoses in a population with known or suspected CAD [5,6]. DCMR-positive patients who did not undergo invasive angiography within 90 days frequently sustained hard cardiac events. Interestingly, the number of patients who sustained early events was relatively high compared to results of the recently published COURAGE and BARI-2D trials which compared invasive vs. conservative management of stable CAD [21,22]. However, there are certain methodological differences between these trials and our study. First, all patients in the above mentioned trials had to have angiographically proven significant coronary stenosis as an inclusion criterion. In the present study, however, patients were classified with regard to inducible ischemia on the myocardial level. Thus, generalization from these trials to the present patient population is limited. Second, subgroup analyses from COURAGE and BARI-2D showed that outcome is worse with complex CAD and high extent of inducible ischaemia, and that early revascularisation in addition to optimal medical therapy was better than optimal pharmacological therapy alone [3,23]. Since early revascularization is likely to improve outcome in these high-risk patients, the pivotal role of cardiac imaging as an arbiter in clinical decision making is further corroborated. In our study patients with early events had a significantly greater extent of ischemia suggesting that an early referral to invasive angiography may be advisable in this patient group. Prior studies using SPECT [24] and echocardiography [25] demonstrated that the extent and severity of stress inducible ischemia is associated with a worse outcome, however, similar data using DCMR testing is still limited.

The vast majority of DCMR-negative patients in our study did not undergo invasive angiography during follow-up time. Similar to SPECT imaging and stress echocardiography, a normal DCMR has generally been associated with a hard annual cardiac event rate of ≈ 1% [10,11,26,27]. Data from randomized trials proved that the low rate of cardiac events in patients with negative stress examinations cannot be improved by revascularization as indicated in current guidelines [1,28]. These patients can be safely treated initially with medical therapy and should only be investigated further if their symptoms cannot be controlled. The impetus for DCMR-driven management has been data from a previously published study dealing with the prognostic value of DCMR and demonstrating a warranty period of two years in case of a negative DCMR test result [11].

In our study 10 percent of the patients with a negative DCMR were subsequently referred for invasive angiography largely owing to recurrent anginal symptoms. In 52 percent revascularization was performed indicating that these patients may have been misclassified as DCMR-neg. The overall "false-negative" rate in our study, however, was low. Previous studies using stress echocardiography have shown that chest pain in the absence of identifiable wall motion abnormalities represents an independent predictor of future cardiac events and should be considered in the interpretation of a normal examination [29]. In addition, it most likely also reflects clinical practice since physicians facing a patient with uncontrolled symptoms are more likely to refer for invasive angiography based on their clinical judgment regardless of the results of prior non-invasive testing. Furthermore, the sensitivity of stress inducible wall motion abnormalities as a marker of ischemia is known to be slightly lower compared to myocardial perfusion imaging techniques. Thus, the addition of perfusion imaging during high dose dobutamine may be helpful in detecting patients with ischemia [4]. Nevertheless, all DCMR-neg patients who underwent invasive angiography without revascularization did not sustain any hard cardiac events supporting the high negative predictive value of the test.

### Limitations

Delayed enhancement (DE) images were not acquired for the purpose of this study. The presence and extent of DE has already been demonstrated to carry independent prognostic value [30,31]. Recently, the combination of CMR vasodilator stress myocardial perfusion and DE was shown to provide complementary prognostic implication for cardiac events [32]. Thus, with regard to prognostication the addition of DE to DCMR may be beneficial in certain patient populations. Visual analysis of invasive angiography by experienced interventional cardiologists was used to determine the degree of coronary luminal narrowing. Quantitative coronary angiography (QCA) is often used as a reference standard in clinical trials, but its usage during routine coronary angiography is rather limited though it may be performed to assist planning of a revascularization procedure. The aim of the present study, however, was to define the role of DCMR testing within a widely seen clinical scenario.

## Conclusions

In our study we demonstrated that a DCMR based management strategy can be used as a reliable gatekeeper to invasive procedures or to substantiate the decision to proceed with medical treatment. Thus, DCMR provides a directive for appropriate and profound clinical management of patients with suspected and known CAD.

## Abbreviations

CAD: Coronary Artery Disease; CMR: Cardiovascular Magnetic Resonance; DCMR: Dobutamine cardiovascular Magnetic Resonance; EF: Ejection Fraction; LV: Left Ventricle; SD: Standard Deviation; IWMA: Inducible Wall Motion Abnormality.

## Competing interests

Bernhard Schnackenburg is an employee of Philips Health Care; Hamburg, Germany.

## Authors' contributions

RG carried out CMR examinations, collected follow up information and drafted the manuscript. CJ carried out CMR examinations and helped with the revision of the manuscript. RM carried out CMR examinations. TH carried out CMR examinations and collected follow up information. BS participated in the study design and helped with the revision of the manuscript. SK carried out CMR examinations. CK carried out and analyzed invasive angiography. EF participated in the study design and helped with the revision of the manuscript. IP conceived of the study, carried out CMR examinations and helped with the revision of the manuscript. All authors read and approved the final manuscript.
